# Observing the morphology of single-layered embedded silicon nanocrystals by using temperature-stable TEM membranes

**DOI:** 10.3762/bjnano.6.99

**Published:** 2015-04-15

**Authors:** Sebastian Gutsch, Daniel Hiller, Jan Laube, Margit Zacharias, Christian Kübel

**Affiliations:** 1 IMTEK, Albert-Ludwigs-University of Freiburg, Georges-Köhler Allee 103, 79110 Freiburg, Germany; 2 Karlsruhe Nano and Micro Facility (KNMF) and Institute of Nanotechnology (INT), Karlsruhe Institute of Technology (KIT), Hermann-von-Helmholtz-Platz 1, 76344 Eggenstein-Leopoldshafen, Germany

**Keywords:** electron irradiation damage, energy-filtered transmission electron microscopy, membrane, plane view, silicon nanocrystals, size control, size distribution

## Abstract

We use high-temperature-stable silicon nitride membranes to investigate single layers of silicon nanocrystal ensembles by energy filtered transmission electron microscopy. The silicon nanocrystals are prepared from the precipitation of a silicon-rich oxynitride layer sandwiched between two SiO_2_ diffusion barriers and subjected to a high-temperature annealing. We find that such single layers are very sensitive to the annealing parameters and may lead to a significant loss of excess silicon. In addition, these ultrathin layers suffer from significant electron beam damage that needs to be minimized in order to image the pristine sample morphology. Finally we demonstrate how the silicon nanocrystal size distribution develops from a broad to a narrow log-normal distribution, when the initial precipitation layer thickness and stoichiometry are below a critical value.

## Introduction

Si nanocrystals (Si NC) are interesting for applications in third generation photovoltaics [1–2], light emitting diodes [3–4], lasers [5], but are also envisioned to be used as non-volatile memories [6–10]. The optical and electrical properties of ensembles of Si NCs are strongly influenced by the structural properties such as size distribution, separation distance and density regardless of the application [11–13]. Moreover, there is compelling evidence of multiple exciton generation in adjacent Si NCs with almost ideal quantum efficiencies which is explained in terms of space-separated quantum cutting due to strong coupling between neighboring Si NCs [14–18]. However, clear structural insight on the Si NC size distribution, separation distance and shape is missing to date, largely due to the complexity of the measurement. The distribution of Si NC sizes is routinely obtained by conventional cross-section transmission electron microscopy (TEM) [19–20], but its evaluation is tedious and areal densities and Si NC position and distance to each other cannot be derived with reasonable confidence. Direct attempts have been made to probe size and density of Si NCs by atomic force microscopy [21]. TEM tomography [22–23] and atomprobe tomography [24–25] were also applied, but these methods probe only a very small volume, may be affected by preparation artifacts and require highly sophisticated equipment, enormous computational effort and time. A faster and easier approach to measure the Si NC size, position and density is the use of in-plane energy-filtered TEM (EFTEM) as was demonstrated for Si NCs formed by low energy Si ion implantation [10,26], plasma-enhanced chemical vapor deposition (PE-CVD) [27] or evaporation [28] followed by a high temperature annealing. The bottleneck in such measurements is the low TEM plane view specimen preparation yield when ultrathin layers are concerned. We circumvent this issue in this work by using nanometer thin, free standing silicon nitride membranes to allow for plane view EFTEM analysis without further sample preparation. Thin layers of Si-rich silicon oxynitride (SRON) can be deposited directly on these membranes that also withstand the high temperature annealing that is needed to induce phase separation and crystallization of the Si NC layers. In contrast to the above mentioned ion implantation, deposition processes allow for sharp interfaces between two confining silicon oxide (SiO_2_) layers. Here, we investigate ultrathin SRON layers by using plane view EFTEM and discuss possible pitfalls in both sample preparation and TEM imaging. Finally, we demonstrate how the thickness and stoichiometry of a SRON layer affects the Si NC size distribution, shape and areal density.

## Experimental

We used 5 nm silicon nitride TEM support grids as a substrate (TEMwindows, SN100-A05Q33A). The layers were prepared by PE-CVD the details of which can be found elsewhere [29]. After layer deposition, the samples were postprocessed by high-temperature annealing. A list of all samples including the relevant processing parameters is available in Table 1. The layer stoichiometries were determined by X-ray photoelectron spectroscopy [29–30]. Please note that we sandwiched the SRON layer between two 2 nm SiO_2_ films to mimic somewhat the SiO_2_ barrier layer in our superlattice approach [19]. The SiO_2_ film thickness is a trade-off between contrast quality during the EFTEM investigation and a reduction of the SiO surface loss during annealing [31] that is discussed later on in this paper in more detail. In fact, we do not observe significant Si surface loss when we use 10 nm thick embedding SiO_2_ layers [32], but the plane view contrast to image Si nanoparticles is strongly reduced due to the largely overlapping plasmon loss peaks of Si and SiO_2_ centered at 17 eV and 24 eV respectively. Please note that the deposition rates of all sublayers were determined in a separate preliminary experiment by using ellipsometry. The EFTEM has been carried out using an image aberration corrected FEI Titan 80-300 microscope operated at 300 kV, equipped with a Gatan 863 Tridiem Imaging Filter and a US1000 slow-scan CCD camera. EFTEM images were acquired with a 5 eV energy slit centered at an energy loss of 17 eV (i.e., the Si plasmon loss energy).

**Table 1:** List of TEM membrane samples fabricated within this work.

sample name	membrane type	active layer	annealing

S1	5 nm SiN	2 nm SiO_2_/4.5 nm SiO_0.64_/2 nm SiO_2_	1150 °C, N_2_, 1 h
S2	20 nm SiO_2_	—	—
S3	5 nm SiN	10 nm SiO_2_	1100 °C, N_2_, 1 h
S4	5 nm SiN	2 nm SiO_2_/10 nm SiO_0.93_/2 nm SiO_2_	1100 °C, Ar^a^
S5	5 nm SiN	2 nm SiO_2_/10 nm SiO_0.93_/2 nm SiO_2_	1100 °C, N_2_^a^
S6	5 nm SiN	2 nm SiO_2_/4.5 nm SiO_0.93_/2 nm SiO_2_	1100 °C, N_2_^a^
S7	5 nm SiN	2 nm SiO_2_/3.5 nm SiO_0.93_/2 nm SiO_2_	1100 °C, N_2_^a^
S8	5 nm SiN	2 nm SiO_2_/3.5 nm SiO_0.85_/2 nm SiO_2_	1100 °C, N_2_^a^
S9	5 nm SiN	2 nm SiO_2_/3.5 nm SiO_0.64_/2 nm SiO_2_	1100 °C, N_2_^a^

^a^annealing was carried in a ramp-up/ramp-down mode with no intentional temperature hold step (see main text for explanation).

## Results and Discussion

### Electron irradiation damage

**Figure 1:**
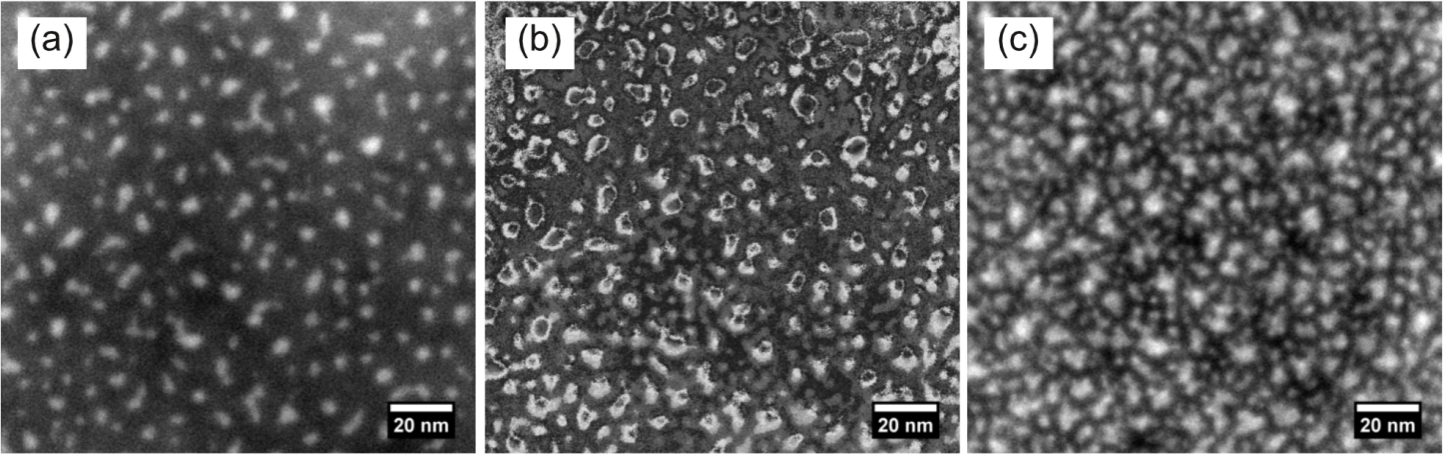
EFTEM images of S1: (a) image in fresh area, (c) after about 10 min exposure to an intense electron beam and (b) XOR image of (a) and (c) highlighting the change between the two as denoted by white regions. Nanoparticles obviously grow and even new ones are created.

**Figure 2:**
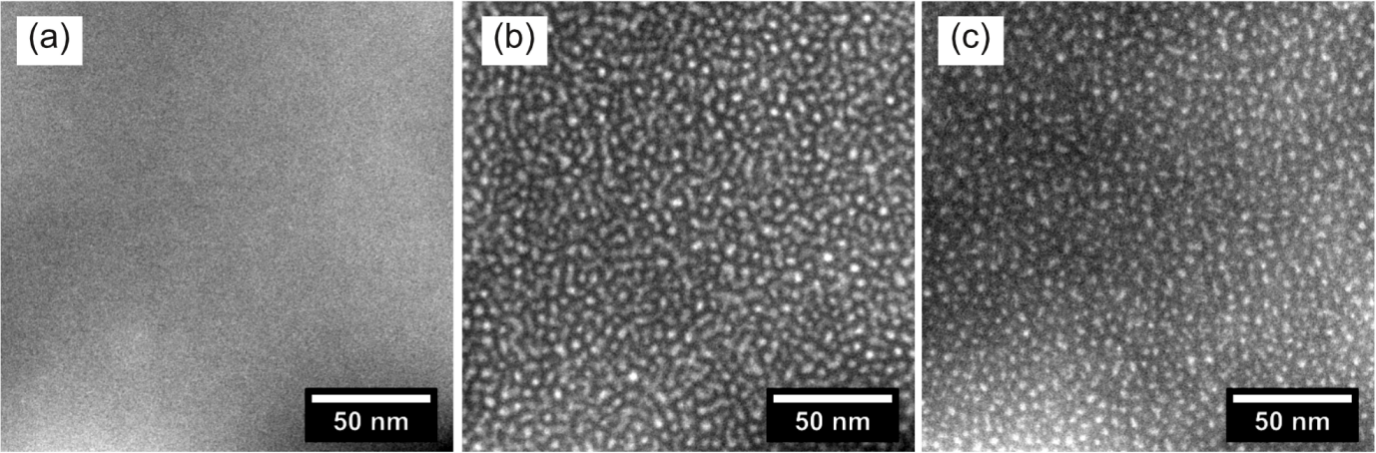
EFTEM images of references samples S2 and S3: (a) S2 irradiated with a low dose, (b) S2 irradiated with a high dose and (c) S3 irradiated with a high dose. After low dose irradiation no Si particles are observed (cf. panel (a)), whereas high doses lead to a dense formation of tiny Si nanoparticles (cf. panels (b) and (c)).

First of all, we focus on a more general observation which mainly concerns the thin film instability during the imaging process. In Figure 1a, a TEM micrograph of sample S1 is presented. In the energy-filtered imaging, the Si particles are clearly visible as white areas. The image has been obtained using low-dose conditions with a total dose of about 5.6 C/cm². In Figure 1c, the dose amounts to about 4.5 × 10^2^ C/cm² in the same area. The particles have significantly grown and new particles appeared, which is clearly visualized in the XOR image of Figure 1b, where image changes are indicated by white. We therefore decided to investigate two plain SiO_2_ films, a SiO_2_ reference TEM membrane and a SiN membrane with PECVD SiO_2_ on top (samples S2 and S3, cf. Table 1). As shown in Figure 2, the creation of Si particles also was observed to take place in pure SiO_2_ and we estimated a threshold irradiation dose of about 16 C/cm². Please note that our calculated threshold dose is in close agreement with literature data [33]. The intense electron beam may cause the breaking of Si–O bonds accompanied by the creation of volatile O_2_ molecules, which is supported by the observation of defect creation in SiO_2_ after electron irradiation [34] and a SiO_2_ thickness dependence on the hole drilling time when exposed to an intense electron probe [35]. Another possible explanation is certainly a preferential sputtering or knock-on of oxygen [36–38]. A common way to reduce the electron beam damage is to lower the operating voltage of the TEM. Hence, we reduced the operation voltage to 80 kV, but did not observe any significant differences in the beam irradiation damage. While the true beam damage mechanism is subject to further investigation, it can be concluded that it is important to take images below the threshold dose, when dielectric films containing Si NCs are investigated.

### Silicon loss and out-diffusion during segregation annealing

Another issue that arises directly from inspection of Figure 1a is the low areal density of sample S1 of only 0.8 × 10^12^ NC/cm². This is a reduction of more than 50% compared to the value of sample S5 treated with a much lower thermal budget (cf. Table 1 and below in Table 2). Lower Si excess concentrations and smaller SRON thicknesses resulted in even lower areal densities. The results suggest that the single Si NC layer is subject to a loss of excess Si during the annealing process. Oxidation of excess Si by some species in the annealing ambient is unlikely because of the highly purified inert gases used during the annealing process. This is further supported by a control experiment, in which we annealed an oxide free Si wafer under the same annealing conditions. An unintentional oxide growth below 1 nm SiO_2_ thickness was estimated by ellipsometry. On the other hand, we need to consider Si diffusion towards the surface followed by a molecular desorption process [31]. The diffusion of Si in SiO_2_ is known to be mediated by co-diffusion of SiO molecules and hence strongly depends on the nature of the Si/SiO_2_ interface [39]. The diffusion length *L*_Si_ of Si can be calculated by 
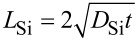
, where *D*_Si_ represents the diffusion constant of Si in SiO_2_. Using the values from literature [39] for a 1 h annealing, *L*_Si_ is determined to be around 2.6 nm which is larger than the 2 nm capping SiO_2_ even though the heating and cooling ramps were not considered. Although the Si surface loss rate is not exactly known, Si out-diffusion and emanation may well explain the observed effects. The diffusion length can be reduced by shortening the annealing time and lowering the annealing temperature. Hence short furnace annealing processes with fast temperature ramps and a peak temperature of 1100 °C are used for all following samples.

### The role of annealing ambient

**Figure 3:**
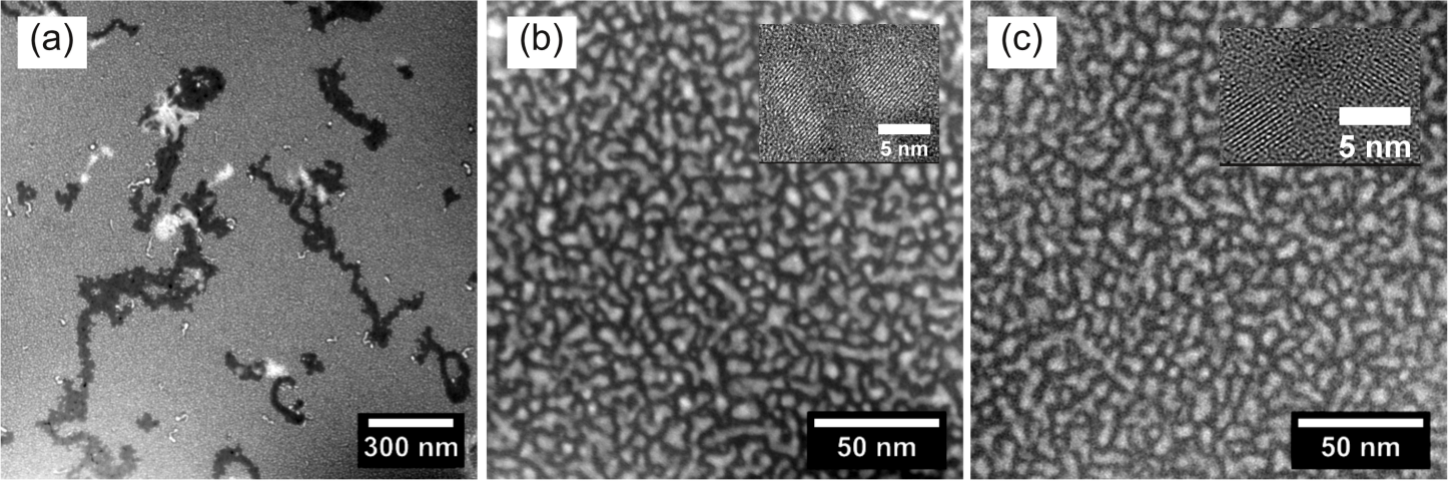
EFTEM images of S4 (a,b) and S5 (c): (a) overview image of S4 illustrating severe sample damage caused by Ar annealing, (b,c) smaller area to show that the morphology in the undamaged regions are fairly similar for both Ar and N_2_ annealing.

It is well known that N2 annealing is usually reducing interface defects and hence leads to higher photoluminescence intensities as compared to Ar annealing [40–41]. Therefore, 10 nm SiO_0_*_._*_93_ films were annealed in Ar and N_2_ (samples S4 and S5) atmosphere and compared by plane-view EFTEM that is presented in Figure 3. As can be seen at lower magnification (Figure 3a), the Ar-annealed sample exhibits a high density of surface defects. It appears that part of the layer has been removed (darker regions). These surface damages are absent for the N_2_-annealed sample, which is very homogeneous across the whole sample area. Similar effects have been reported for Ar-annealed SiO_2_ thin films on Si and were ascribed to out-diffusion of SiO molecules [31]. Therefore, it can be concluded that annealing in N_2_ hampers the Si out-diffusion. However, the undamaged microstructures (cf. Figure 3b and Figure 3c) are very similar for both annealings. From high resolution TEM (see insets of Figure 3b and Figure 3c) and electron diffraction, we found that both samples feature a high degree of crystallinity as is corrobated by detailed Raman studies [20,42]. However, the Si NC shape is not spherical at all. Due to the minimization of Gibbs free energy, a spherical shape is expected, which is limited by the possibility of atomic rearrangement. Since the phase separation is completed within a few seconds due to diffusion of oxygen [43], the nanoparticle growth and shaping can only be achieved through the diffusion of Si within SiO_2_, which is extremely low at the used thermal budget. Longer and higher temperature annealings would certainly lead to larger amounts of spherical particles with a reduced density due to late-stage coarsening [27,44].

### Silicon nanocrystal size distribution and areal density

**Figure 4:**
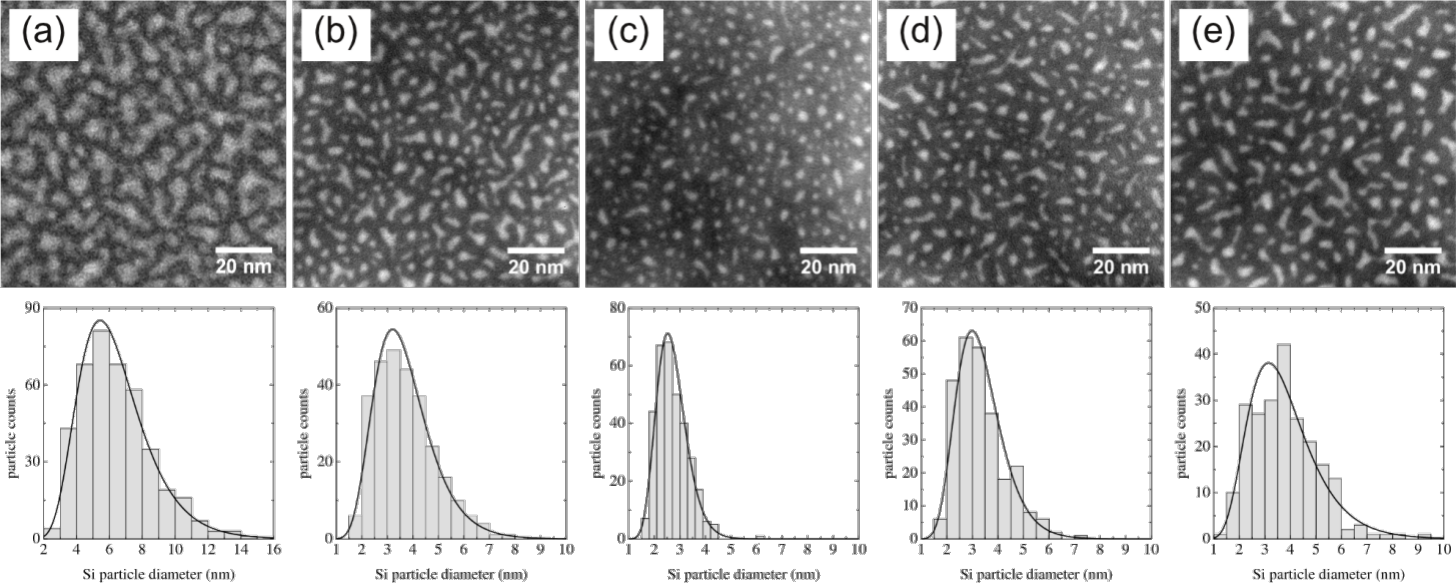
EFTEM images and corresponding Si NC size distributions of S5-S9: (a) S5 (10 nm SiO_0.93_), (b) S6 (4.5 nm SiO_0.93_), (c) S7 (3.5 nm SiO_0.93_), (d) S8 (3.5 nm SiO_0.85_), (e) S9 (3.5 nm SiO_0.64_).

Once the annealing conditions and TEM routines have been specified, it is straightforward to investigate the influence of the SRON layer thickness and stoichiometry on density, size and shape of the Si NCs. In Figure 4 a series of EFTEM images is presented for samples S5 to S9. We first consider Figure 4a–c in which the SRON thickness is reduced from 10 nm to 3.5 nm for a fixed layer stoichiometry of SiO_0.93_. A transition from irregular and large towards spherical and smaller precipitates is clearly observed. Owing to the excellent contrast, the images can be evaluated by image processing software such as ImageJ [45] in order to analyze the particle distribution. The individual particle areas have been assigned to a circle of the same area which allows for the characterization of all particles through a single parameter. The diameter distributions are shown below the corresponding EFTEM images in Figure 4. All distributions can be fitted excellently by a log-normal distribution. The results strongly reflect the ability to control the Si NC size by geometrical one-dimensional confinement of the SRON layers. Furthermore, the influence of the SRON stoichiometry on Si nanoparticle formation is demonstrated in Figure 4c–e. Interestingly, increasing the Si excess exhibits a similar effect on the Si NC size and shape as the SRON thickness increase. The transition from clustering to spinodal-like decomposition [46–48] is obviously a sensitive function of the SRON thickness as well as of the stoichiometry. Due to the one dimensional geometrical confinement imposed by the SiO_2_ barriers, the effective excess Si available for particle formation is reduced and hence spinodal growth sets in at higher SRON thicknesses [49]. As the Si excess in the SRON layer is increased, the SRON thickness threshold for spinodal decomposition is shifted to smaller values [49–50], a fact that is experimentally demonstrated in Figure 4.

The EFTEM studies are summarized in Table 2, where the maximum of the diameter distribution as well the particle areal density *A*_NC_ and the particle area fill fraction are given. Please note that such detailed information as provided by the plane-view method here, is not available in cross-section TEM imaging [19–20,29]. The areal particle density is increased and the Si NC diameter is decreased, when the SRON layer thickness is reduced as expected. However, increasing the Si excess reduces the areal density. The idea of the superlattice approach is to control the Si NC size and density independently by variation of the SRON thickness or stoichiometry respectively [19]. The results presented here are in contrast to the assumptions of this idealized superlattice approach. The reason is apparently that at high Si excess concentrations, larger Si regions form as is indicated by the increased average Si NC diameter. Finally, it must be noted that a certain amount of Si still appears to be lost by Si out-diffusion during the optimized annealing that effectively decreases the particle size.

**Table 2:** Extracted parameters from the EFTEM analysis, *d*_NC_ indicates the maximum of the log-normal distribution fit, whereas *A*_NC_ is the Si NC areal density.

sample name	SRON layer	*d*_NC_ (nm)	*A*_NC_ (NC/cm²)	area fill fraction

S5	10 nm SiO_0.93_	5.4	(1.13 ± 0.02) × 10^12^	40.8%
S6	4.5 nm SiO_0.93_	3.2	(2.43 ± 0.05) × 10^12^	28.8%
S7	3.5 nm SiO_0.93_	2.6	(2.88 ± 0.06) × 10^12^	17.7%
S8	3.5 nm SiO_0.85_	3.0	(2.32 ± 0.05) × 10^12^	22.3%
S9	3.5 nm SiO_0.64_	3.2	(1.95 ± 0.04) × 10^12^	24.6%

## Conclusion

In conclusion, we have demonstrated an approach using ultrathin TEM membranes and EFTEM imaging as a very versatile tool to study the morphology of Si NC ensembles in contrast to the limitation imposed by cross sectional TEM investigations [19–20,29]. We proved that low electron doses have to be used in order to image the real Si NC structure since higher irradiation doses lead to undesired growth and expansion of the SiNCs. Furthermore, we have shown that for these samples, large surface damage occurs when annealing in Ar atmosphere, whereas this damage is minimized in N_2_ atmosphere. SiNC size distributions and areal densities were measured for a variety of sample parameters such as initial SRON thickness and stoichiometry. It is demonstrated that SiNC size and density cannot be controlled individually by changing the thickness or stoichiometry of the SRON layer. On the one hand the average SiNC size is controllable by the SRON thickness, on the other hand an increase of the Si excess concentration results in a larger SiNC formation with a reduced areal density. Please note that the observed trends are certainly representative for the given sample parameters, but may still deviate from the individual layer structure in a superlattice.
